# Efficacy of Central Neck Dissection for Clinically Node-Negative Papillary Thyroid Carcinoma: Propensity Scoring Matching

**DOI:** 10.3389/fendo.2019.00172

**Published:** 2019-03-27

**Authors:** Byung Joon Yoo, Chang Myeon Song, Yong Bae Ji, Ji Young Lee, Hae Jin Park, Kyung Tae

**Affiliations:** ^1^Department of Otolaryngology-Head and Neck Surgery, College of Medicine, Hanyang University, Seoul, South Korea; ^2^Department of Radiology, College of Medicine, Hanyang University, Seoul, South Korea; ^3^Department of Radiation Oncology, College of Medicine, Hanyang University, Seoul, South Korea

**Keywords:** papillary thyroid carcinoma, total thyroidectomy, central neck dissection, propensity score matching, recurrence, survival

## Abstract

**Objectives:** The utility of prophylactic central neck dissection (pCND) for papillary thyroid carcinoma (PTC) is still controversial. Although the procedure may reduce locoregional recurrence, it is associated with a high rate of postoperative complications. The aim of this study was to evaluate the role of pCND in patients with PTC.

**Materials and Methods:** From January 1995 to April 2011, the records of 477 patients who underwent total thyroidectomy with or without pCND for clinically node-negative PTC measuring < 4 cm were retrospectively reviewed. Of these, 341 patients had undergone pCND with total thyroidectomy and 136 patients did not undergo pCND. The clinicopathologic characteristics, surgical outcomes, complications, recurrence, and survival were analyzed using propensity score matching, using age, sex, tumor size, extrathyroidal extension, and radioactive iodine ablation as covariates to minimize selection bias.

**Results:** At baseline, there was no significant difference in sex, age, and multiplicity and bilaterality of the cancer between the two groups. However, extrathyroidal extension was more common and tumor size larger in patients who underwent pCND. For the propensity score-matched analysis, two matched groups, each comprising 135 patients, were generated. After propensity score matching, the significant differences observed at baseline between the two groups disappeared. The postoperative complication rate did not differ between the two groups. Recurrence occurred in 4 patients (2.96%) who had undergone pCND and in 2 patients (1.48%) who did not undergo pCND (*P* = 0.684). The recurrence-free survival curves did not differ between the two groups.

**Conclusion:** The efficacy of pCND in total thyroidectomy for clinically node-negative PTC is limited, and pCND is not recommended for these patients.

## Introduction

In the last 2 decades, the incidence of thyroid cancer has shown a sharp and continuous increase all over the world. In Korea, notably, the incidence of thyroid cancer has increased by 24% per year recently, and it became the most prevalent cancer in 2009 (1).

Papillary thyroid carcinoma (PTC) accounts for 80 to 90% of all thyroid carcinomas. Patients with PTC have a good prognosis, with overall disease-specific mortality rates < 10% at the 20-year follow-up (2–4). However, PTC is characterized by a high incidence of cervical lymph node metastasis, which is associated with local recurrence and distant metastasis, and also has a negative effect on survival (4–9).

Prophylactic central neck dissection (pCND) has been performed in patients with PTC to reduce lymph node recurrence and provide more accurate staging to facilitate decision-making regarding adjuvant radioactive iodine (RAI) ablation (4, 10–12). However, pCND is associated with a high rate of postoperative complications and so far, there is no conclusive evidence for improved survival and low recurrence rate after pCND (4, 13–17). In fact, it remains controversial whether pCND is necessary in patients with clinically node-negative (cN0) PTC. Therefore, in this study, we evaluated the clinical efficacy of pCND in the treatment of node-negative PTC, using propensity score matching to minimize selection bias and the effect of confounding factors that might affect the oncologic outcome.

## Materials and Methods

We retrospectively reviewed the medical records of 477 patients who underwent total thyroidectomy with or without pCND for clinically lymph node-negative PTC by the conventional transcervical approach from April 1995 to April 2011 in a tertiary hospital. Of these 477 patients, 341 (71.49%) underwent pCND with total thyroidectomy (bilateral pCND in 188 cases and unilateral pCND in 153 cases) and 136 (25.51%) patients underwent total thyroidectomy only without pCND. All thyroidectomy and pCND was performed by two authors. The decision of pCND depended on the status of disease and surgeon's and patient's preference. In bilateral pCND, pretracheal, prelaryngeal, and bilateral paratracheal lymph nodes were removed, and in unilateral pCND, pretracheal, prelaryngeal, and ipsilateral paratracheal lymph nodes were removed. The pCND group was defined as having the operation record about the pCND, and the number of harvested lymph nodes was more than two. We excluded patients with tumors measuring >4 cm; major extrathyroidal extension (ETE); suspicious lymph node metastasis on pre-operative physical examination, ultrasonography, or computed tomography; recurrent disease; distant metastasis; and other types of thyroid cancer. We also excluded patients who had undergone lobectomy or concomitant lateral neck dissection with total thyroidectomy. All patients provided written informed consent for their medical records to be reviewed, and the protocol of the study was approved by the institutional review board of Hanyang University Guri Hospital.

All patients had undergone ultrasonography (US) and computed tomography (CT) to evaluate the status of cervical lymph node metastasis, and had been diagnosed with PTC preoperatively by fine needle aspiration cytology (FNAC). Clinically, node-negative status was defined as the absence of suspicious lymph nodes on US and CT images regardless of cytological confirmation. Minimal ETE was defined as the extension of the primary tumor through the thyroid capsule, wherein the tumor remained confined to the perithyroidal soft tissue or the strap muscle kin the postoperative pathologic findings. Maximal ETE was defined as the spread of the tumor to surrounding structures such as the larynx, trachea, esophagus, recurrent laryngeal nerve, subcutaneous soft tissue, skin, internal jugular vein, or carotid artery. Postoperative radioactive iodine (RAI) ablation was performed in some selected patients with minimal ETE or cervical lymph node metastasis, and higher risk histologic features. The RAI ablation dose ranged from 30 to 100 mCi.

TNM staging of the cancers was performed according to the American Joint Committee on Cancer/International Union for Cancer Control (AJCC/UICC) 7th edition. All patients underwent fiberoptic flexible laryngoscopy on the day before surgery and on the day after surgery. Permanent vocal cord paralysis was defined as vocal cord paralysis persisting for more than 6 months. Hypoparathyroidism was defined as the presence of serum parathyroid hormone levels below 15 pg/ml. Permanent hypoparathyroidism was defined as the continuation of low levels of hormone for >6 months. Recurrence was defined as the development of new abnormal structural lesions identified on physical examination, neck US, or CT and confirmed pathologically by FNAC.

Clinicopathologic characteristics such as sex, age, tumor size, the presence of multiple tumors, lymphovascular, or perineural invasion and TNM stage, surgical outcomes including operative time, the number of resected lymph nodes, postoperative complications, RAI ablation, and recurrence and survival rates were analyzed and compared, using propensity score matching, between patients who had undergone pCND and those who had not. Propensity score matching was performed to overcome patient selection bias and to adjust for confounding factors that might affect the comparison between the two groups. Five covariates—age, sex, tumor size, ETE, and RAI ablation—were used as variables and propensity scores were calculated using logistic regression analysis with Statistical Package for the Social Sciences version 20.0 (SPSS Inc, Chicago, IL, USA). Pairwise matching was performed without replacement, using nearest neighbor matching.

Statistical analysis was performed using Statistical Package for the Social Sciences version 20.0 (SPSS Inc, Chicago, IL, USA). The Chi-square test or Fisher's exact test was used for comparison of categorical variables, and the *T*-test, Mann–Whitney *U*-test and Wilcoxon signed rank test were used for continuous variables. The survival rate was calculated using the Kaplan-Meier method and *p* < 0.05 was considered statistically significant.

## Results

Clinicopathologic data for the baseline cohort of 477 PTC patients are presented in Table 1. The mean tumor size was higher in patients with pCND than in patients without pCND. The incidences of minimal ETE, lymphovascular invasion, T classification, and radioactive iodine ablation dose were also significantly higher in the pCND group than in the non-pCND group. The mean number of lymph nodes removed was 6.35 ± 5.77 (range 2–40).

**Table 1:** Baseline clinicopathological characteristics of patients with papillary thyroid carcinoma with or without prophylactic central neck dissection.

**Variable**	**Total thyroidectomy with CND(+) (*n* = 341)**	**Total thyroidectomy with CND(-) (*n* = 136)**	***P***
**Sex**	341	136	0.958
Male	52	21	
Female	289	115	
Age (years)	49.70 ± 12.33	48.07 ± 12.55	0.194
**Pathologic subtype**			0.285
Classic PTC	341 (100%)	135 (99.3%)	
Follicular variant	0	1 (0.7%)	
Tumor size, mm	11.77 ± 8.81	8.98 ± 6.44	0.000
Minimal extrathyroidal extension	144 (42.23%)	33 (24.26%)	0.000
Multiplicity	85 (24.93%)	27 (19.85%)	0.238
Bilaterality	72 (21.11%)	25 (18.38%)	0.503
Lymphovascular invasion	58 (32.04%, *n* = 181)	15 (15.96%, *n* = 94)	0.004
Perineural invasion	7 (4.79%, *n* = 146)	1 (1.43%, *n* = 70)	0.442
**T classification**			0.034
T1	194 (56.89%)	96 (70.59%)	
T2	26 (7.62%)	10 (7.35%)	
T3	121 (35.48%)	30 (22.06%)	
T4	0 (0%)	0 (0%)	
**N classification**			NA
Nx		136 (100%)	
N0	218 (63.93%)		
N1a	123 (36.07%)		
**Stage**			0.000
I	215 (63.05%)	110 (80.88%)	
II	6 (1.76%)	8 (5.88%)	
III	120 (35.23%)	18 (13.24%)	
IV	0 (0%)	0 (0%)	
**CND**			
Unilateral	153 (44.87%)		
Bilateral	188 (55.13%)		
Radioactive iodine ablation	299 (87.68%)	120 (88.23%)	0.868
Radioactive iodine ablation dose (mCi)	103.45 ± 56.77	77.31 ± 54.75	0.000
Number of lymph node removed	6.35 ± 5.77 (2–40)		

*CND, Central neck dissection; NA, Non-applicable*.

Propensity score matching generated two matched groups with 135 patients each. The clinicopathologic data after propensity score matching are shown in Table 2. In the propensity score-matched cohort, there were no significant differences in age, sex, tumor size, ETE, lymphovascular invasion, perineural invasion, multiplicity, TNM staging, and the rate and dose of radioactive iodine ablation between the groups.

**Table 2:** Clinicopathological characteristics of patients with papillary thyroid carcinoma with or without central neck dissection after propensity score matching.

**Variables**	**Total thyroidectomy with CND (*n* = 135)**	**Total thyroidectomy without CND (*n* = 135)**	***P***
**Sex**	135	135	0.732
Male	19	21	
Female	116	114	
Age (years)	49.39 ± 10.74	48.24 ± 12.42	0.417
Tumor size, mm	8.01 ± 5.04	8.97 ± 6.46	0.177
**Pathologic subtype**			1.0
Classic PTC	135 (100%)	134 (99.3%)	
Follicular variant	0	1 (0.7%)	
Minimal extrathyroidal extension	34 (25.19%)	32 (23.7%)	0.777
Multiplicity	34 (25.19%)	27 (20%)	0.308
Bilaterality	27 (20%)	25 (18.52%)	0.758
Lymphovascular invasion	18 (24.32%, *n* = 74)	15 (15.96%, *n* = 94)	0.175
Perineural invasion	2 (3.23%, *n* = 62)	1 (1.43%, *n* = 70)	0.600
**T classification**			0.507
T1	105 (77.78%)	96 (71.11%)	
T2	1 (0.74%)	10 (7.41%)	
T3	29 (21.48%)	29 (21.48%)	
T4	0 (0%)	0 (0%)	
**N classification**			NA
Nx		135 (100%)	
N0	102 (75.56%)		
N1a	33 (24.44%)		
**TNM staging**			0.120
I	103 (76.3%)	109 (80.74%)	
II	0 (0%)	8 (5.93%)	
III	32 (23.7%)	18 (13.33%)	
IV	0 (0%)	0 (0%)	
**CND**			
Unilateral	59 (43.7%)		
Bilateral	76 (56.3%)		
Number of lymph node removed	6.44 ± 5.12 (2–23)		

*CND, Central neck dissection; NA, Non-applicable*.

In the propensity score-matched cohort, there was no significant difference in the operative time between the two groups. Complications such as hematoma, temporary vocal cord palsy, and permanent hypoparathyroidism also did not differ significantly between the groups (Table 3). The rate of transient hypoparathyroidism was higher in the pCND group than in the non-pCND group, but this difference was not statistically significant (*P* = 0.051). The mean of postoperative serum thyroglobulin was not different between the two groups.

**Table 3:** Comparison of operative times, postoperative complications, and radioactive iodine ablation between the propensity score-matched groups.

**Variables**	**Total thyroidectomy with CND (*n* = 135)**	**Total thyroidectomy without CND (*n* = 135)**	***P***
Operation time (min)	170.7 ± 47.56	163.37 ± 42.62	0.183
Hematoma	5 (3.7%)	5 (3.7%)	1.00
Transient hypoparathyroidism	75 (55.56%)	58 (42.96%)	0.051
Permanent hypoparathyroidism	5 (3.7%)	3 (2.22%)	0.722
Transient vocal cord palsy	1 (0.74%)	4 (2.96%)	0.370
Permanent vocal cord palsy	0 (0%)	0 (0%)	
Radioactive iodine ablation	110 (81.48%)	119 (88.15%)	0.127
Radioactive iodine ablation dose (mCi)	88.18 ± 58.55	77.31 ± 54.75	0.148
Postoperative serum thyroglobulin (ng/ml)	0.96 ± 1.57	1.13 ± 2.13	0.474

*CND, Central neck dissection*.

Structural recurrence occurred in 4 cases (2.96%) and 2 cases (1.48%) in the pCND group and the non-pCND group (*P* = 0.684), respectively, in the propensity score-matched cohort (Table 4). The mean follow-up period was 92 and 90 months, and there was no statistically significant difference between the groups. The recurrence site was the ipsilateral lateral lymph node in all 4 cases in the pCND group. Among patients with only total thyroidectomy, 2 recurrences occurred in the central compartment lymph node (Table 4). In two of six patients with recurrence, serum thyroglobulin level increased at the time of recurrence.

**Table 4:** Comparison of recurrence in the propensity score-matched groups.

**Variables**	**Total thyroidectomy with CND (*n* = 135)**	**Total thyroidectomy without CND (*n* = 135)**	***P***
Follow up period (month)	92.19 ± 38.47	90.1 ± 43.95	0.679
Recurrence	4 (2.96%)	2 (1.48%)	0.684
Time to recurrence (month)	71.5 ± 27.21	49.5 ± 20.51	0.355
**Recurrence Site**			
Central lymph node	0	2	
Lateral lymph node	4	0	

*CND, Central neck dissection*.

**Figure 1:**
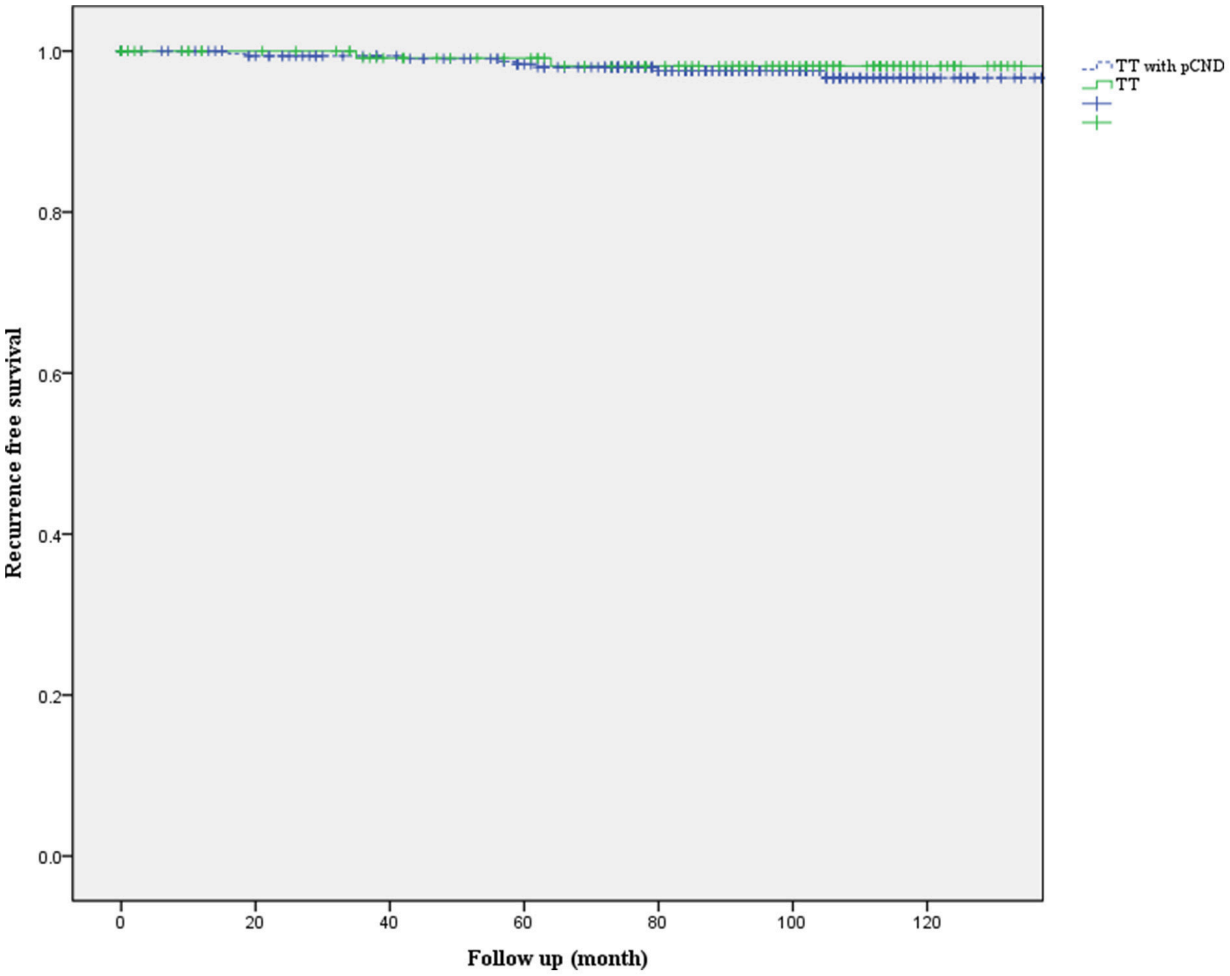
Kaplan-Meier recurrence-free survival curves of patients with papillary thyroid carcinoma who underwent total thyroidectomy with or without prophylactic central neck dissection (pCND) (*P* = 0.571).

Kaplan-Meier recurrence-free survival curves were not statistically different between the two groups (*P* = 0.571, Figure 1). The 5-year recurrence-free survival rate was 97.2% in patients with pCND and 98.1% in patients without pCND.

## Discussion

Prophylactic CND in clinically node-negative PTC remains controversial. The reason in support of pCND are that it decreases the risk of recurrence and reoperation and allows more precise staging by identifying occult lymph node metastases, which can affect decisions about postoperative RAI ablation (4, 11–13, 18–22). However, opponents insist that pCND does not lower the recurrence rate or increase the survival rate and that it increases the occurrence of postoperative complications such as hypoparathyroidism and vocal cord palsy (16, 17, 23, 24).

Guidelines on pCND have gone back-and-forth over the last decade. Prophylactic CND was routinely recommended for patients with cN0 PTC regardless of tumor size in the 2006 American Thyroid Association (ATA) guidelines (25). However, in the 2009 and 2015 ATA guidelines, the indications for pCND was confined to advanced primary tumors (T3 or T4) (26, 27). It was not recommended for cN0 non-invasive T1 or T2 PTC (26, 27).

As per the 2014 British Thyroid Association (BTA) guidelines, central compartment neck dissection is not recommended for patients without clinical or radiological evidence of lymph node involvement and who have all of the following characteristics: classical type PTC, <45 years, unifocal tumor, ≤4 cm, and no ETE on ultrasonography (28). As per the 2018 NCCN guidelines, pCND is not recommended if the cervical lymph nodes are clinically negative (29, 30).

The purpose of this study was to evaluate the efficacy of pCND in total thyroidectomy for clinically node-negative PTC < 4 cm by using propensity score matching to reduce the selection bias. Propensity score matching analysis is increasingly being used to evaluate the impact of various treatments. In this study, significant differences between the two groups observed in the baseline cohort disappeared after propensity score matching. In the two propensity score-matched groups, there was no significant difference in operative time and complications, except transient hypoparathyroidism. Transient hypoparathyroidism tended to be higher in patients with pCND although it did not reach the statistical significance (*P* = 0.051). Recurrence rate and recurrence-free survival also did not differ between the pCND group and non-pCND group. These results suggest that pCND has no role in reducing the recurrence rate and in improving the survival rate. In terms of recurrent site, in the propensity score-matched cohort the pCND group had no recurrence in the central compartment while two patients of the non-CND group were recurred in the central compartment. However, if we look at the baseline data, of 341 patients who underwent pCND with total thyroidectomy, there were 8 recurrences (6 recurrences in the lateral neck and 2 recurrences in the central neck). Two patients had recurrences in the central compartment although they received pCND. Actually, there was no statistical significance in the comparison of recurrence rate in the central compartment between the two groups of this study. Therefore, it is difficult to say that pCND reduces recurrence in the central compartment.

Zetoune et al. conducted a meta-analysis comparing 396 patients with PTC who had undergone pCND and 868 PTC patients who had undergone thyroidectomy only. The results showed that pCND did not reduce local recurrence, similar to the results of this study (31). Another study by Kim et al. also investigated 11,569 patients with clinically node-negative PTC. Of 11,569 patients, 8,735 (75.5%) had undergone pCND, and pCND did not decrease the risk of locoregional recurrence significantly (*P* = 0.392). However, the occurrence of postoperative complications such as temporary vocal cord palsy (*P* = 0.001), temporary hypoparathyroidism (*P* < 0.001), and permanent hypoparathyroidism (*p* < 0.001) was significantly higher in the pCND group (17).

In contrast, a meta-analysis study performed by Zhao et al. revealed that pCND with total thyroidectomy reduced the risk of local recurrence significantly although the incidence of temporary and permanent hypoparathyroidism and temporary recurrent laryngeal nerve injury increased (10).

Several studies have reported conflicting results on the efficacy of pCND in PTC (4, 9–11, 13, 17, 22, 29, 31, 32). However, based on the results of this study and previous studies, pCND does not seem to be necessary in the surgical treatment of clinically node-negative PTC, especially for PTC < 4 cm and no major ETE although it might be very difficult to make a solid recommendation. Our findings might provide one piece of evidence supporting the 2015 ATA guidelines on pCND.

There are several limitations of this study. First, it is a retrospective non-randomized observational study; therefore, there is inherent selection bias although propensity score-matching analysis was performed to minimize this bias. Second, the study included relatively small numbers of patients. A very large sample size is necessary to determine small differences in recurrences between the two groups. Third, most tumors were < 2 cm; tumors larger than 2 cm only comprised 11.5% of the study cohort. Therefore, it is difficult to determine the role of pCND clearly in PTC sized 2–4 cm. Fourth, the number of lymph node removed was 2 or 3 in some patients who underwent pCND. It might be related with inadequate pCND although the adequate number of lymph nodes removed to define the CND has not been determined. Further prospective studies with larger sample size and including larger tumors are necessary to overcome the limitations of this study.

## Conclusion

Based on the aforementioned findings, the efficacy of prophylactic CND might be limited in PTC. Therefore, pCND might not be recommended for PTC, especially for PTC < 4 cm and no major ETE.

## Data Availability

The datasets generated for this study are available on request to the corresponding author.

## Author Contributions

BY: analysis and interpretation of data, writing of draft, final approval, agreement. CS and YJ: acquisition and interpretation of data, article revision, final approval, agreement. JL: acquisition of data, article revision, final approval, agreement. HP: interpretation of data, article revision, final approval, agreement. KT: conception and design of the study, interpretation of data, article revision, final approval, agreement.

### Conflict of Interest Statement

The authors declare that the research was conducted in the absence of any commercial or financial relationships that could be construed as a potential conflict of interest.
